# Dural effects of oxidative stress on cardiomyogenesis via Gata4 transcription and protein ubiquitination

**DOI:** 10.1038/s41419-018-0281-y

**Published:** 2018-02-14

**Authors:** Tao Li, Xia Zhang, Kesheng Jiang, Jing Liu, Zhiqiang Liu

**Affiliations:** 1 School of Medicine, Hunan Normal University, Changsha, Hunan 410013 China; 2 Department of Biology, Zhejiang Normal University, Jinhua, Zhejiang 321004 China; 3 Department of Physiology and Pathophysiology, School of Basic Medical Science, Tianjin Medical University, Heping, Tianjin, 300070 China

## Abstract

Oxidative stress generates reactive oxygen species (ROS) that can promote or inhibit cardiac differentiation of stem cells dependent on the intensity of stimuli as well as cellular context in redox and differentiation status. In the current study, we confirmed that suitable intensity of hydrogen peroxide at the formation stage of embryoid bodies (EBs) effectively favored the formation of spontaneously beating cardiomyocytes from P19 embryonal carcinoma cells. Mechanistic studies implicated that extrinsic ROS enhanced the Caspase-mediated degradation of Oct4 and Nanog, two factors that governing pluripotent property. Further experiments suggested that a cohort of Nanog together with histone deacetylase 4 (Hdac4) played a critical role in establishing and maintaining the silent transcriptional status of *Gata4* and *Nkx2.5* in undifferentiated cells. Thus, an impulse of hydrogen peroxide depleted Nanog and Hdac4 via a caspase-dependent manner to ameliorate the repression on *Gata4* and *Nkx2*.5 promoters, thereby generating a persistent activation on cardiac differentiation program. Meanwhile, we found that excessive ROS-activated JNK cascade to facilitate the ubiquitination and subsequent degradation of Gata4 protein. Overall, our results indicate that suitable ROS promotes the activation of Gata4 in transcription, while excessive ROS targets Gata4 protein for proteasome-dependent degradation. Gata4 is an important modulator balancing the promoting and inhibitory effects of oxidative stress on differentiation program of cardiomyogenesis.

## Introduction

Cardiac differentiation is a complicated stepwise process, which is tightly regulated by the chronological integration of transcriptional program and signaling pathways. The precise role of distinct signaling pathway during cardiomyogenesis is dependent on the cell context and the differentiation stage, exhibiting remarkable stage-specific and dose-dependent characteristics. Several signaling pathways have been revealed to be associated with the stepwise process of cardiac differentiation, such as TGF-β, Wnt, and Notch^1^. These signaling pathways exert distinct effect and govern the determination of cell fate during the critical steps of cardiac differentiation. Manipulating signaling pathways can direct the committed differentiation of stem cells to the cardiac lineages, thereby efficiently yielding homogeneous and sufficient number of cardiomyocytes^2, 3^.

Oxidative stress stimulates the production of reactive oxygen species (ROS), a group of highly reactive molecules, which participate in various biological events as an intracellular signal. However, the excessive emergence of ROS also causes cell damage or disorder. Thus, oxidative stress can be beneficial or deleterious, depending on the magnitude and duration of stimuli, and the overall redox context and differentiation status of cells. In adult heart, ischemic injury produces massive ROS, which predisposes cardiomyocytes to chronic dysfunction, damage, and death. On the other hand, the burst of ROS also activates the proliferation and differentiation of endogenous or implanted stem cells to repair the damaged myocardium. Importantly, differentiation of embryonic stem (ES) cells towards cardiomyocytes can be elevated by ROS generated by intracellular NADPH oxidases or from extrinsic supplement at the formation stage of embryoid bodies (EBs)^4–7^. Conversely, free radical scavengers can exert opposite effects on cardiac differentiation of stem cells. However, the precise mechanisms of ROS and downstream signaling transduction in cardiac differentiation remain to be elucidated. Specially, the rationale for a burst of ROS stimulation to modify cardiac gene program is mainly unknown.

P19 embryonic carcinoma cells are multi-potent and can differentiate into derivatives of all three germ layers. A combination of EB formation and DMSO induction can efficiently drive P19 cells to differentiate into cardiac myocytes. In this study, we aim to study the effects of stress-producing stimuli on cardiac differentiation in P19 cells, evaluate whether stress-producing stimuli can promote or impair cardiac differentiation dependent on its intensity and duration, and explore the underlying molecular mechanisms associated with cardiac gene program and epigenetic regulation.

## Results

### Oxidative stress favors cardiomyogenesis of P19 cells

**Fig. 1:**
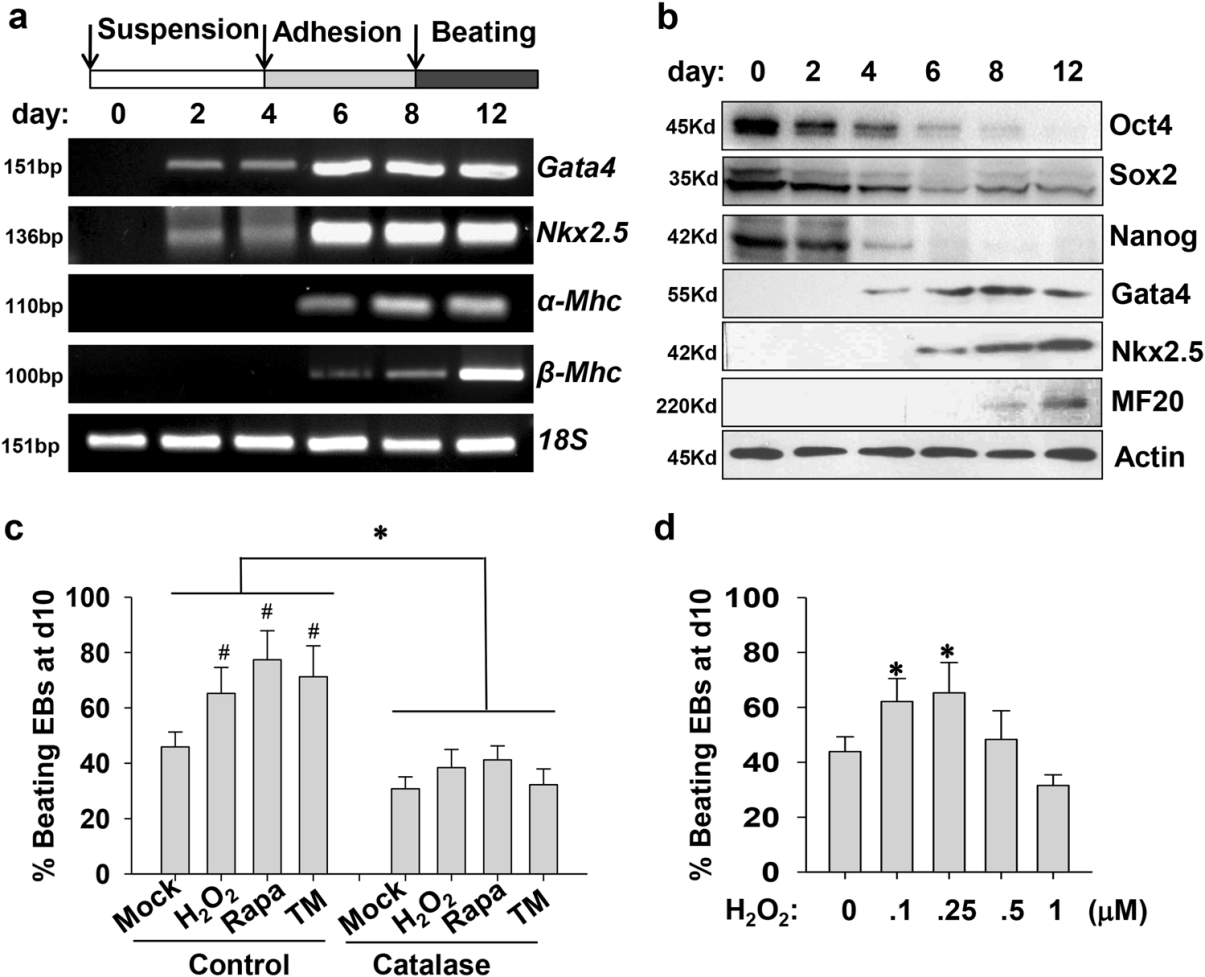
Positive effects of three stress-producing stimuli on cardiac differentiation of P19 cells. **a** Schematic diagram of differentiation protocol with DMSO. P19 cells were cultured in suspension for embryoid body (EB) formation and then were plated for another 4 days in the presence of 1% DMSO. Cells were harvested at indicated days, and extracts were analyzed by RT-PCR to show the expression profile of cardiac transcriptional factors (*Gata4* and *Nkx2.5*) and contractile myosin heavy chain (*α-Mhc* and *β-Mhc*). 18S rRNA expression was used as an internal control. **b** Representative western blot analysis shows the expression kinetics of pluripotent genes and cardiac-specific genes in protein level during the process of cardiac induction. **c** Stimulation of cardiomyogenesis by three stress-producing stimuli. P19-cell-derived EBs were treated at day 4 of cardiac induction in suspension with 100 nM H_2_O_2_, 20 nM Rapa, or 0.5 μg/ml TM. After 24 h, the medium was changed and EBs were plated for adhesion induction. The percentage of contracting EBs was counted at day 10 among these groups. The pretreatment of ROS scavenger catalase (200 U/ml, Sigma) for 0.5 h at day 4 destroyed the differentiation-promoting effects of H_2_O_2_, Rapa, and TM. Data obtained from three independent experiments were shown as means ± SD. **d** The dosage-dependent effects of H_2_O_2_ treatment (0, 100, 250, 500, 1000 nM at day 4) on cardiac differentiation of P19 cells. Differentiation efficiency of cardiomyogenesis was evaluated as the percentage of EBs with beating areas. Values are expressed as means of three independent experiments ± SD (*p *<* 0.05; #*p* < 0.01 vs. the control)

P19 cells were firstly incubated with DMSO in suspension, allowing cells to aggregate and form EBs, subsequently, EBs were plated for adherent growth with DMSO for another 4 days. During the late stage of cardiac differentiation, stem cells became capable of expressing cardiac contractile protein genes *α-Mhc* and *β-Mhc* (Fig. 1a). Spontaneously contracting cardiomyocytes initially emerged from day 8–10 and progressively expanded in number. In protein levels, Gata4 and Nkx2.5 proteins were first detected from day 4 or 6 of cardiac induction. Cardiac myosin heavy chain MF-20 expression initially appeared at day 8 and gradually increased over the following periods (Fig. 1b). Transcriptional factors, Oct4 and Nanog maintaining the pluripotent stem cell phenotype, declined from the early stage of differentiation and rapidly vanished at the late stage (Fig. 1b). Another pluripotent factor Sox2 displayed different degradation manner from Oct4 and Nanog.

As several studies revealed that inducible production of ROS at the EB formation stage can promote cardiac differentiation^8, 9^. Subsequently, we treated P19 cells at day 4 with different stimuli, such as H_2_O_2_, autophagy inducer rapamycin (Rapa), endoplasmic reticulum (ER) stress inducer tunicamycin (TM) at suitable concentrations without significant toxic effects on cell growth and survival. Interestingly, all three stimuli favored cardiomyogenesis of P19 cells, as characterized by increasing amount of beating EBs (Fig. 1c). Comparatively, treatment at the early-differentiation stage can achieve more prominent effects than at the late stage (data not shown). All three stimuli were reported to evoke oxidative stress at suitable concentrations^10, 11^. And the differentiation-promoting effects of all three stimuli could be abolished by the pretreatment of a free radical scavenger, catalase (Fig. 1c). Excessive H_2_O_2_ was detrimental to the generation of spontaneously contractile cardiomyocytes (Fig. 1d). Obviously, a short-time occurrence of exotic stress at the early-differentiation stage can trigger a permanent alteration in differentiation program of cardiac induction, thereby facilitating the eventual generation of contracting cardiomyocytes.

### Oxidative stress activates p38 and JNK signaling

**Fig. 2:**
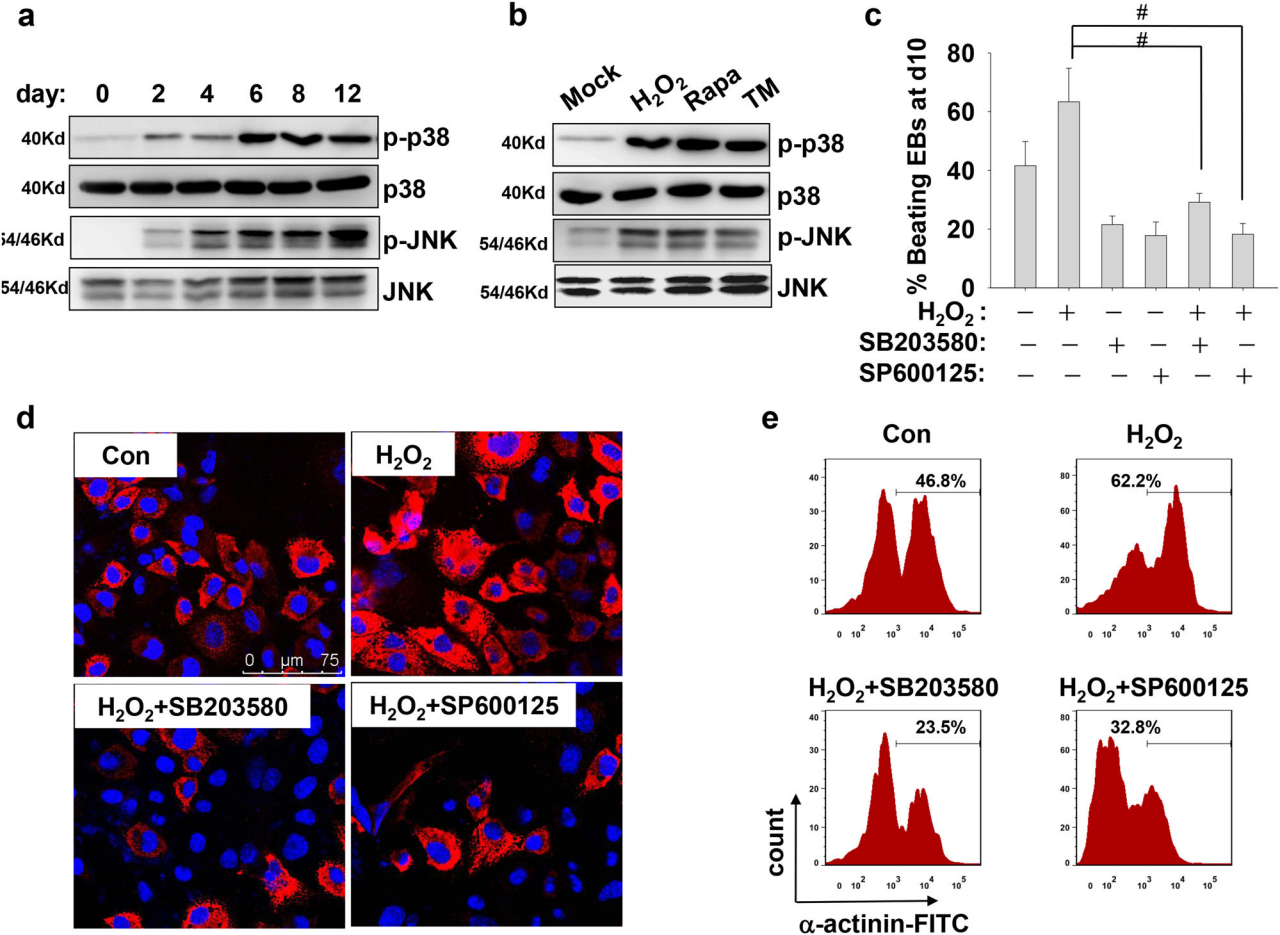
p38 and JNK activation are required for ROS-enhanced cardiomyogenesis. **a** Representative western blot analysis shows the activation of p38 and JNK pathways during cardiac induction. The cell lysates were extracted from differentiating P19 cells at the indicated time points and subjected for western blot with antibodies against the total and phosphorylated protein levels of p38 and JNK kinases. **b** EBs at day 4 of differentiation were treated with H_2_O_2_, Rapa, and TM. Two hours later, cells were collected and subjected for western blot to detect the levels of phosphorylated p38 and JNK kinases. **c** EBs were administrated with 100 nM H_2_O_2_ 0.5 h after addition of specific inhibitors (10 μM SB203580, 10 μM SP600125) at day 4 of differentiation. Percentages of spontaneously contracting EBs at day 10 of differentiation were determined (*p < 0.05). **d** Immunofluorescence images show cardiomyocytes derived from P19 cells, which were treated with H_2_O_2_ plus p38 or JNK inhibitors as described above. Differentiating cells at day 12 were staining with a monoclonal antibody against sarcomeric α-actinin (Red). Nuclei were counterstained with Hoechst33342 (Blue). **e** Flow cytometry assay shows the sarcomeric α-actinin expression in differentiated P19 cells at day 12 treated with H_2_O_2_ in present or absent of the p38 and JNK inhibitors

Next, we investigated the temporal activation of p38 MAPK and JNK signaling. As shown in Fig. 2a, the activation of JNK and p38 MAPK during the differentiation procedure shared a similar activation pattern with biphasic kinetics. High levels of phosphorylated JNK and p38 mainly appeared and maintained at the late stage of differentiation. The complementary effect of H_2_O_2_, Rapa, and TM directly elevated the phosphorylation levels of p38 and JNK kinases (Fig. 2b). Further experiments validated that the activation of p38 and JNK kinases was required for efficiency differentiation. The supplement of specific inhibitors for p38 (SB203580) or JNK (SP600125) at day 4 of cardiac induction all impeded the differentiation efficiency, as evaluated by calculating the percentage of beating EBs at day 10 of induction (Fig. 2c). Meanwhile, treatment with SB203580 or SP600125 at the early stage greatly interrupted H_2_O_2_-enhanced cardiomyogenesis, as evaluated by calculating the percentage of beating EBs (Fig. 2c), by immunofluorescence for sarcomeric protein α-actinin (Fig. 2d), or by flow cytometry for FITC-labeled α-actinin staining (Fig. 2e). Thus, our results implicated that oxidative stress activates p38 MAPK and JNK signaling to promote cardiomyogenesis.

### Oxidative stress alters the expression of pluripotent and cardiac-specific genes

**Fig. 3:**
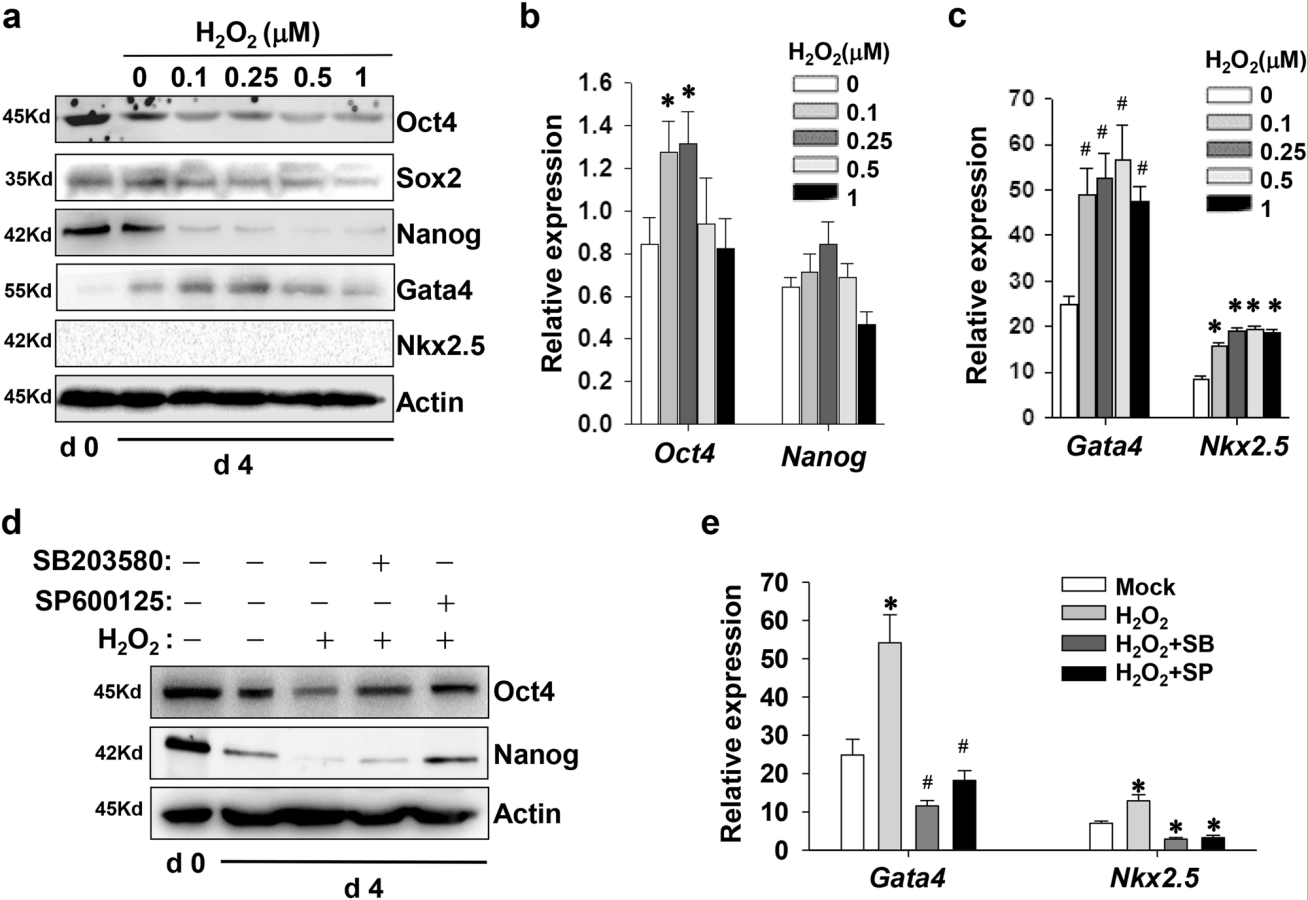
H_2_O_2_ treatment affects the expression of pluripotent and cardiac-specific genes. **a** Representative western blot analysis shows the effects of H_2_O_2_ on the expression of pluripotent and cardiac-specific genes in protein levels. EBs at day 4 of differentiation were treated with increasing dosages of H_2_O_2_. Twenty-four hours later, cells were collected and subjected for western blot. **b**, **c** Real-time PCR assays estimate the effects of H_2_O_2_ on the expression of pluripotent and cardiac-specific genes in transcript levels. **d** The blockade of p38 and JNK pathways interrupts the H_2_O_2_-induced decrease in the protein levels of Oct4 and Nanog. EBs were treated with 10 μM SB203580 or 10 μM SP600125 0.5 h prior to addition of 100 nM H_2_O_2_, and cultured for another 24 h. **e** p38 and JNK inhibition block the H_2_O_2_-upregulation of cardiac-specific genes in mRNA levels (*p < 0.05; #*p* < 0.01 vs. the control)

P19 cells were treated with increasing amounts of H_2_O_2_ at day 4 of cardiac induction, and then were subjected for real-time PCR and immunoblotting assay. As shown in Fig. 3a, H_2_O_2_ treatment during the early stage significantly decreased the expression of Oct4 and Nanog, which is a feature of cell exit from the pluripotent status. As the relative expression of *Oct4* and *Nanog* messenger RNA (mRNAs) remained stable or became slightly upregulated, the effects of H_2_O_2_ treatment might attribute to accelerated degradation of Oct4 and Nanog (Fig. 3b). By comparison, H_2_O_2_ treatment indeed enhanced the amounts of *Gata4* and *Nkx2.5* transcripts (Fig. 3c). SB203580 and SP600125 partially reversed the H_2_O_2_-induced decrease of Oct4 and Nanog in protein levels. And SP600125 was more effective in rescuing Nanog expression than SB203580 (Fig. 3d). However, high concentrations of H_2_O_2_ were invalid to enhance the protein amount of Gata4, although there were accompanied by an augmented *Gata4* mRNA expression (Fig. 3a, b). Blockage of p38 and JNK cascades completely abolished the upregulation of *Gata4* and *Nkx2.5* expression (Fig. 3e).

### Oxidative stress activates caspase-3 to deplete Oct4 and Nanog

**Fig. 4:**
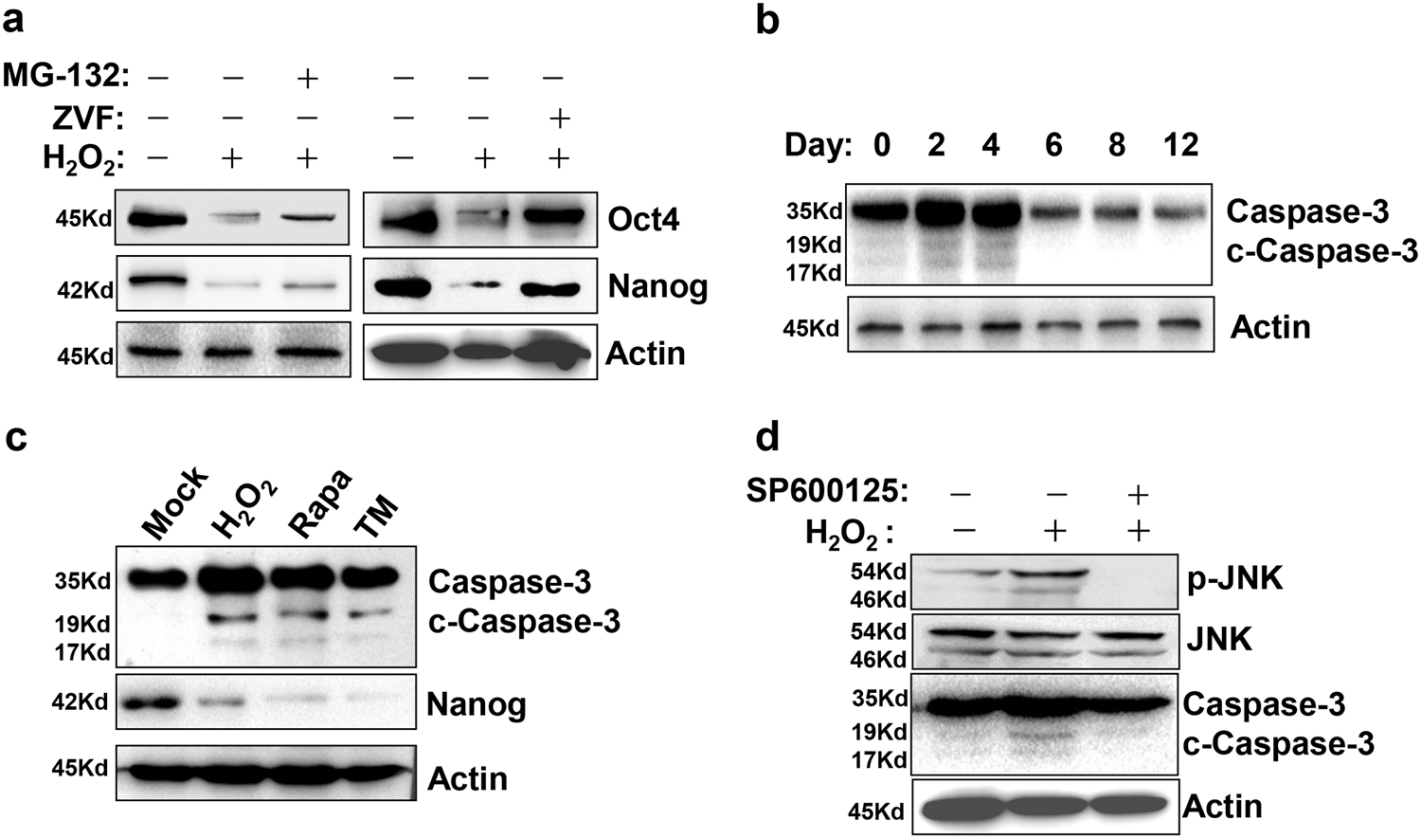
H_2_O_2_ treatment degrades Oct4 and Nanog via a caspase-dependent manner. **a** Caspase inhibition protects Oct4 and Nanog from H_2_O_2_-induced degradation. EBs at day 4 of differentiation were treated with 20 µM Z-VAD-FMK or 200 nM MG-132 0.5 h prior to addition of 100 nM H_2_O_2_. Twenty-four hours later, cells were collected and subjected for western blot. **b** Representative western blot analysis shows the activation profile of caspase-3 during the whole process of cardiac differentiation. **c** EBs at day 4 of differentiation were treated with H_2_O_2_, Rapa, and TM. Two hours later, cells were collected and subjected for western blot to detect the cleavage of caspase-3. **d** The pretreatment of JNK inhibitor SP600125 decreased H_2_O_2_-induced cleavage of caspase-3

To determine the precise pathway mediating the degradation of Oct4 and Nanog, P19 cells in the early differentiation were treated with H_2_O_2_ at the presence of the proteasome inhibitor MG-132 and the pan Caspase inhibitor Z-VAD-FMK. As shown in Fig. 4a, Z-VAD-FMK rather than MG-132 was capable of rescuing the H_2_O_2_-decreased Oct4 and Nanog. Therefore, caspase-mediating depletion accounted for the degradation of Oct4 and Nanog. In fact, we found that there existed a temporal activation of caspase-3 at the early stage throughout the whole process of cardiac induction (Fig. 4b). The administration of H_2_O_2_, Rapa, and TM induced an intensified generation of cleaved caspase-3 (Fig. 4c). In addition, the induced activation of caspase-3 could be reversed by the blockade of JNK cascade (Fig. 4d).

### Nanog degradation relieves the repression of *Gata4* and *Nkx2.5* transcription

**Fig. 5:**
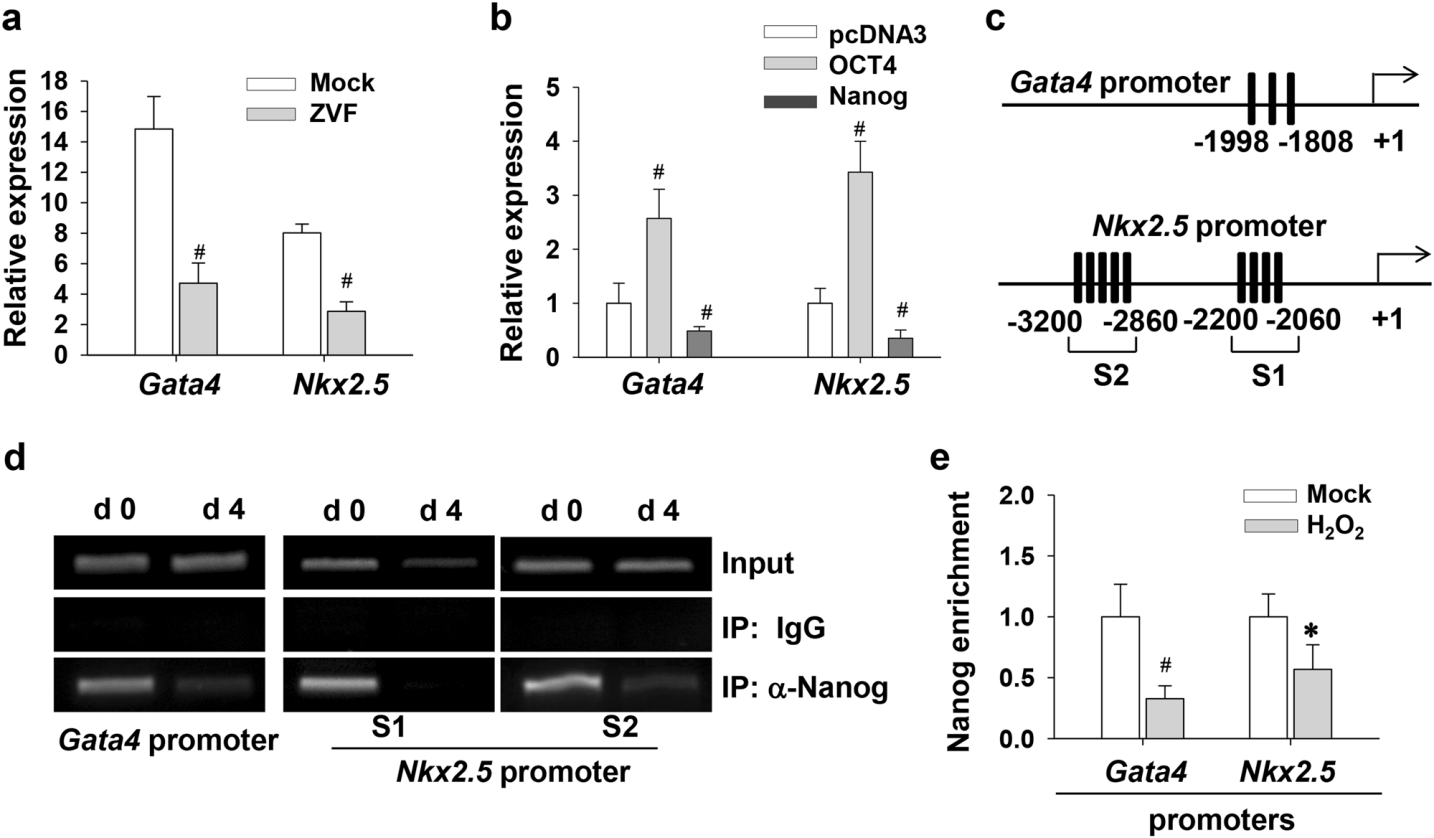
Caspase activation promotes *Gata4* and *Nkx2.5* expression via alleviating the repression of Nanog. **a** Caspase inhibition reduces *Gata4* and *Nkx2.5* expression. EBs at day 4 were treated with 20 µM Z-VAD-FMK or vehicle. Twenty-four hours later, total RNA was extracted from each samples and subjected for real-time PCR assay to examine the expression of *Gata4* and *Nkx2.5*. Date are normalized to the expression of *18S* rRNA and expressed as fold changes relative to the control group. **b** The effects of Oct4 and Nanog on *Gata4* and *Nkx2.5* expression. P19 cells were transfected with plasmids encoding *Oct4* or *Nanog*. After 48 h of transfection, the mRNA levels of *Gata4* and *Nkx2.5* were measured and normalized to *18S* rRNA gene. **c** TAAT-rich regions on the promoters of *Gata4* and *Nkx2.5* genes. Only conserved TAAT sequences among human, mouse, and rat species are showed. **d** ChIP assay was carried out to detect Nanog binding on the *Gata4* and *Nkx2.5* promoters in undifferentiated P19 cells. Input represented 10% of the total input chromatin, and IgG served as a negative control. **e** After exposure to 100 nM H_2_O_2_ for 24 h, differentiating EBs were subjected to ChIP experiments using anti-Nanog antibody. The immunoprecipitated DNA fragments were amplified by real-time PCR for *Gata4* and *Nkx2.5* (S1 region) promoters containing putative Nanog-binding sites. Each bar represents the mean ± SD for triplicate experiments (*p < 0.05; #*p* < 0.01 vs. the control)

Since Z-VAD-FMK blocked the degradation of Oct4 and Nanog, we subsequently investigated the effects of caspase inhibition on *Gata4* and *Nkx2.5* expression. As shown in Fig. 5a, Z-VAD-FMK downregulated the expression of *Gata4* and *Nkx2.5* mRNA at day 4 of differentiation. We further investigated whether the augment of *Gata4* and *Nkx2.5* expression in response to oxidative stress was not a result from Oct4 and Nanog degradation. Unexpectedly, ectopic expression of Oct4 in undifferentiated P19 cells effectively increased the transcripts of *Gata4* and *Nkx2.5* genes (Fig. 5b). Importantly, Nanog overexpression repressed *Gata4* and *Nkx2.5* expression. There exist multiple Nanog-binding TAAT-rich domains on *Gata4* and *Nkx2.5* promoters (Fig. 5c). ChIP assays were conducted with anti-Nanog antibody, and DNA fragments containing the putative Nanog-binding sites were detected. Along with the effect of H_2_O_2_ treatment on Nanog expression, the enrichment of Nanog on the promoters of *Gata4* and *Nkx2.5* genes also decreased after H_2_O_2_ exposure (Fig. 5d). Therefore, our results established a link between Nanog degradation and the transactivation of *Gata4* and *Nkx2.5* genes.

### **Oxidative stress promotes Hdac4 degradation to facilitate the expression of*Gata4*and*Nkx2.5***

**Fig. 6:**
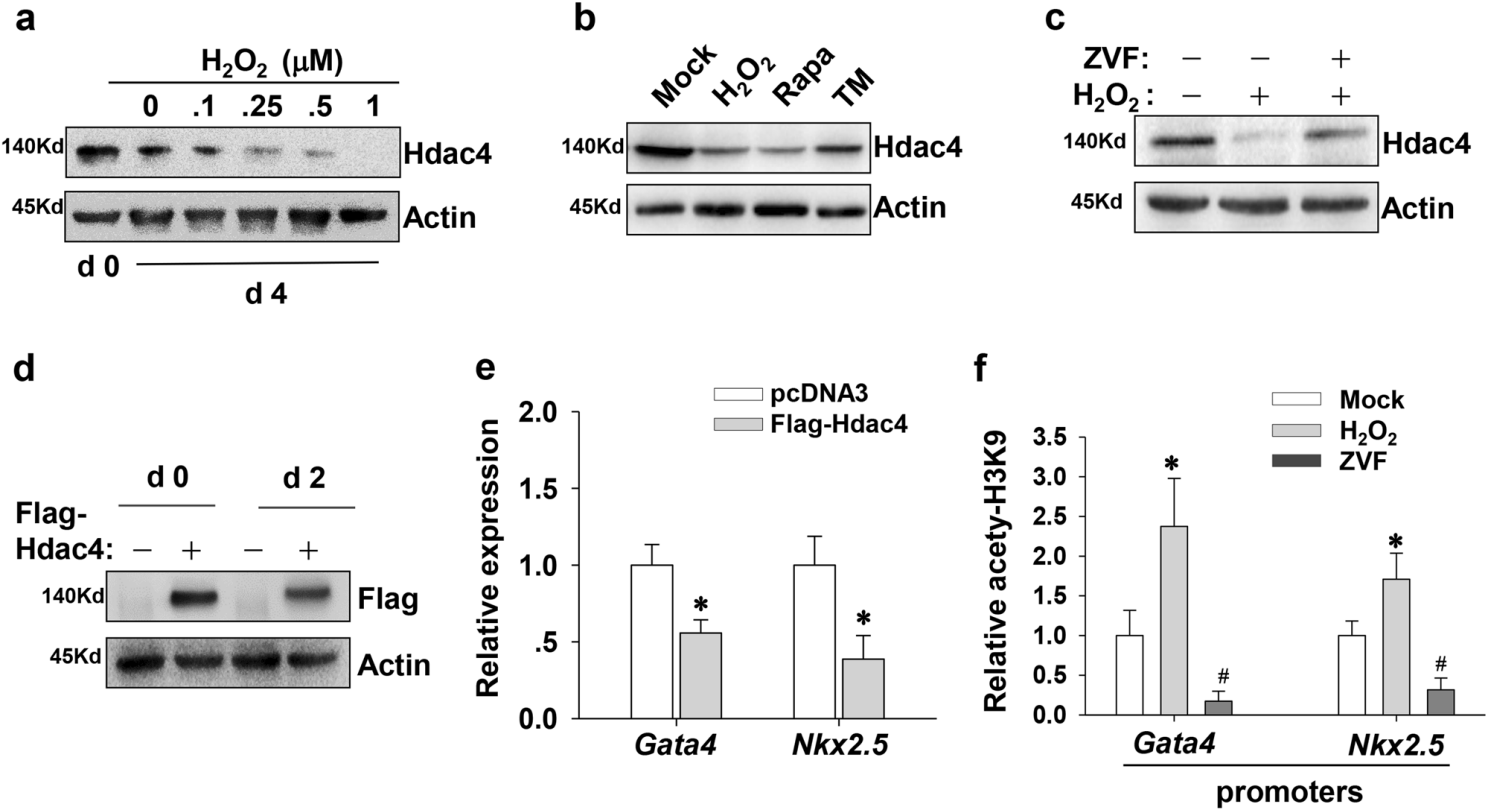
H_2_O_2_ treatment degrades Hdac4 to facilitate *Gata4* and *Nkx2.5* expression. **a** Representative western blot analysis shows the effects of H_2_O_2_ with increasing concentrations on Hdac4 expression. **b** EBs at day 4 of differentiation were treated with 100 nM H_2_O_2_, 20 nM Rapa, or 0.5 μg/ml TM for 24 h. Then cells were collected and subjected for western blot to detect the amount of Hdac4. **c** Caspase inhibition blocks Hdac4 degradation induced by H_2_O_2_. EBs at day 4 of differentiation were treated with 20 µM Z-VAD-FMK before the addition of 100 nM H_2_O_2_. Twenty-four hours later, cells were collected and subjected for western blot. **d** P19 cells were transfected with plasmids encoding *Hdac4*. After transfection, P19 cells were allowed to aggregate and differentiate in the presence of 1% DMSO, and then were subjected for western blot. **e** Hdac4 overexpression hampers the transcriptional activation of *Gata4* and *Nkx2.5*. After transfection with Hdac4 plasmids, P19 cells were induced under suspension culture for 2 days. **f** Caspase inhibition is detrimental to H3K9 acetylation on *Gata4* and *Nkx2.5* promoters during cardiomyogenesis. EBs at day 4 of differentiation were treated with 100 nM H_2_O_2_ or 20 µM Z-VAD-FMK. Twenty-four hours later, cells were collected and ChIP assays were conducted with antibodies against H3K9ac. Immunoprecipitated DNA fragments were amplified by real-time PCR for the promoter regions of *Gata4* and *Nkx2.5* (S1 region). Each bar represents mean ± SD from three independent experiments (*p < 0.05; #*p* < 0.01 vs. the control)

We further exploit whether other substrates of caspase-3 participate in the early-stage differentiation. It is well known that histone deacetylase Hdac4 is a substrate for caspase-3^12,13^. In this study, we examined whether caspase-3 depletes Hdac4 to activate cardiac differentiation. Firstly, we validated that the expression of Hdac4 gradually decreased in response to increasing amount of H_2_O_2_ (Fig. 6a). As expected, Rapa and TM also triggered the degradation of Hdac4 protein (Fig. 6b). Antagonism of caspase activity indeed reversed the inducible degradation of Hdac4 (Fig. 6c). Furthermore, we performed transient transfection with FLAG-Hdac4 plasmids and found that Hdac4 overexpression indeed reduced the upregulation of *Gata4* mRNA during cardiac induction (Fig. 6d, e). Likewise, the transcriptional level of *Nkx2.5* gene was also abrogated by the forced expression of Hdac4 (Fig. 6e). Obviously, Hdac4 degradation was beneficial for the transactivation of *Gata4* and *Nkx2.5* genes. As a result of Hdac4 depletion, H_2_O_2_ administration increased the levels of H3K9 acetylation on *Gata4* and *Nkx2.5* promoters. The interference of Z-VAD-FMK dramatically blemished the acetylation status of *Gata4* and *Nkx2.5* promoters (Fig. 6f). These results further implicate that the expression of cardiac progenitor genes during cardiomyogenesis is tightly linked with Hdac4 downregulation and histone hyper-acetylation at promoter regions.

### **Nanog cooperates with Hdac4 to inhibit*Gata4*and*Nkx2.5*expression**

**Fig. 7:**
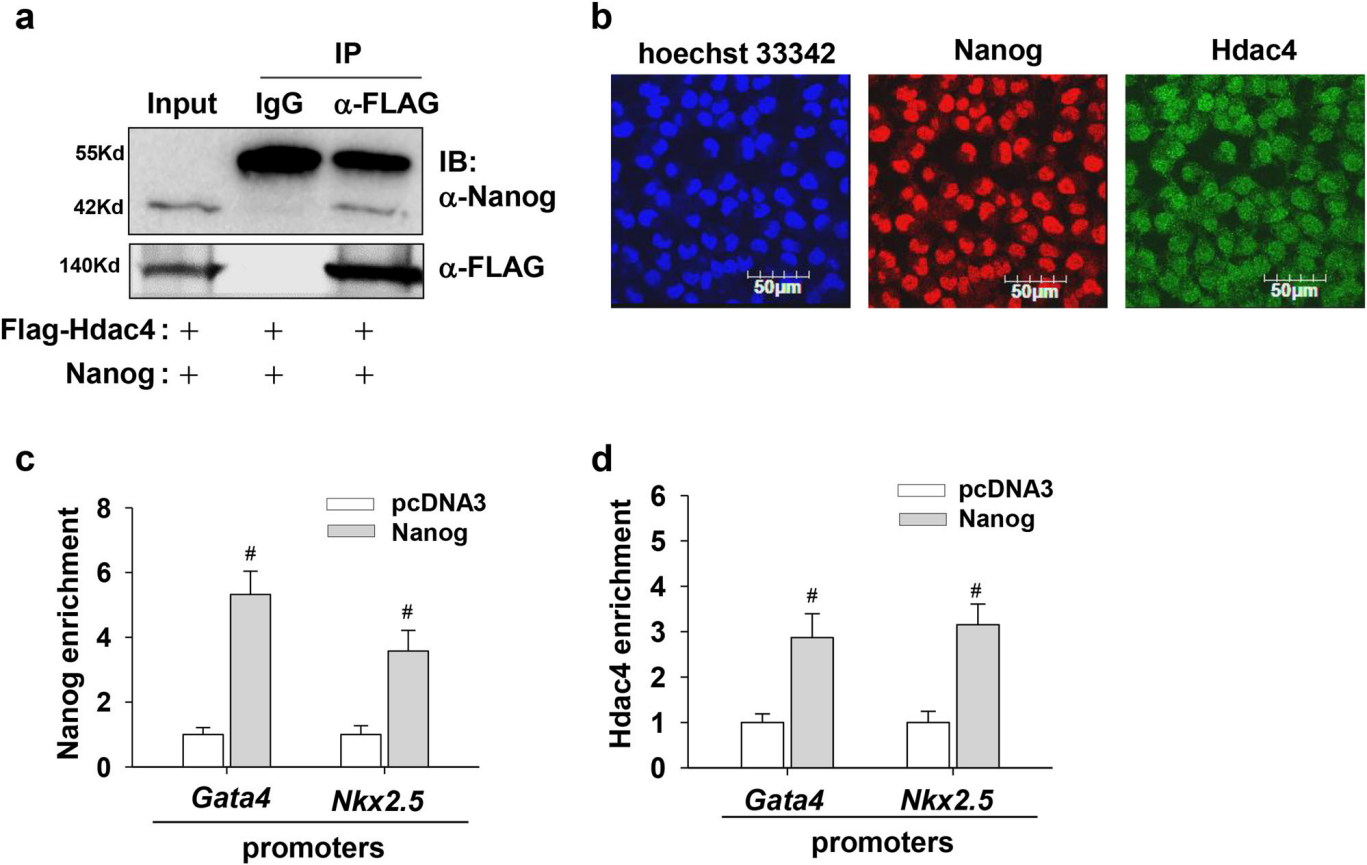
Functional association between Nanog and Hdac4 inhibits *Gata4* and *Nkx2.5* expression. **a** Immunoprecipitation assay shows the functional interaction of Nanog and Hdac4. 293T cells were transfected with expression vectors for Nanog and FLAG-Hdac4. Cell lysates were subjected to immunoprecipitation with anti-FLAG antibody or normal IgG as a negative control, and the precipitates were subjected to immunoblot analysis with anti-Nanog or anti-FLAG antibodies, respectively. Five percent of the whole-cell lysate was loaded for the input control. **b** Immunostaining of undifferentiated P19 cells with specific antibodies shows the subcellular localization of Nanog and Hdac4. Hoechst dye was used for staining nuclei. **c**, **d** Nanog overexpression facilitates the recruitment of Hdac4 onto *Gata4* and *Nkx2.5* promoters. Undifferentiated P19 cells were transfected with plasmids encoding *Nanog*. After 48 h of transfection, cells were collected and ChIP assays were conducted with antibodies against Nanog (**c**) and Hdac4 (**d**). Immunoprecipitated DNA fragments were amplified by real-time PCR for the promoter regions of *Gata4* and *Nkx2.5* (S1 region). Each bar represents mean ± SD from three independent experiments (*p < 0.05; #*p* < 0.01 vs. the control)

Given the similar roles of Nanog and Hdac4 in regulating cardiac-specific genes, we hypothesized there might be a functional interaction between these two proteins. Subsequently, we performed Co-IP studies in HEK293T cells cotransfected with Nanog and FLAG-Hdac4 plasmids. As shown in Fig. 7a, Nanog was co-immunoprecipitated with FLAG-Hdac4, suggesting a direct interaction between these two proteins. Meanwhile, immunofluorescence staining showed that Nanog and Hdac4 both mainly localized in the nucleus of undifferentiated cells (Fig. 7b). In parallel to an enhanced occupation of Nanog onto the promoters of *Gata4* and *Nkx2.5* genes after Nanog overexpression, an augmented amount of Hdac4 protein was also recruited onto the same genomic regions (Fig. 7c, d). It implicates that Nanog can recruit Hdac4 onto *Gata4* and *Nkx2.5* promoters to establish a silent transcriptional status. Taken together with previous findings, our results suggest that there is a functional association of Nanog and Hdac4 in regulating *Gata4* and *Nkx2.5* expression.

### Excessive ROS promotes Gata4 degradation via JNK cascade

**Fig. 8:**
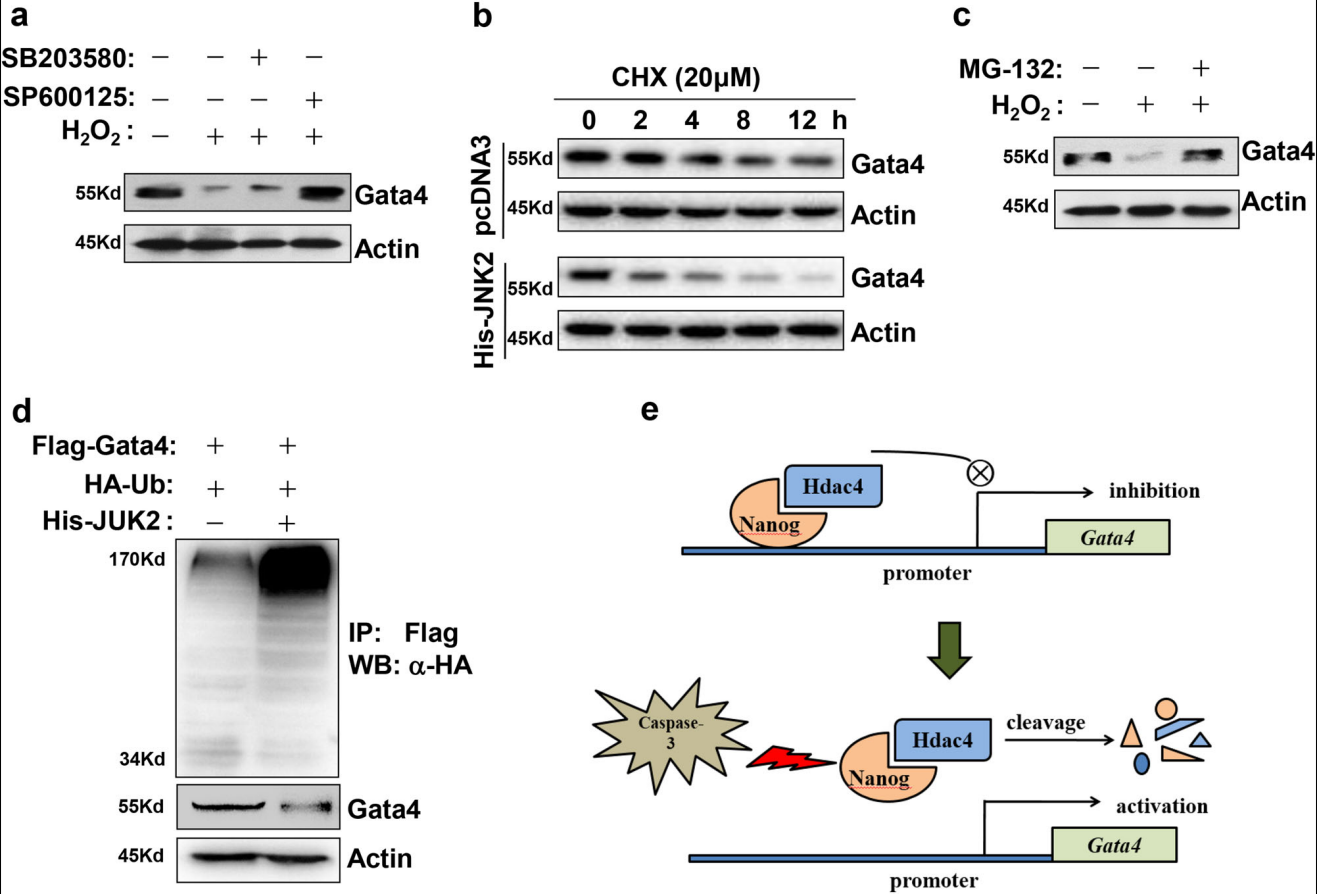
Excessive H_2_O_2_ degrades Gata4 via JNK-activated ubiquitination pathway. **a** JNK inhibition alleviates the repression of excessive H_2_O_2_ on Gata4 protein expression. EBs at day 4 of differentiation were treated with 1000 nM H_2_O_2_ in the presence of JNK or p38 inhibitors. Twenty-four hours later, cells were collected and subjected for western blot to detect Gata4 protein. **b** Accelerated degradation of Gata4 protein under JNK2 overexpression. Twenty-four hours later after transfection with Gata4 plasmid, P19 cells were treated with 20 μM cycloheximide (CHX) for indicated periods. Western blot results show changes of the half-life of exogenous Gata4 protein. **c** Proteasome inhibition reverses Gata4 degradation induced by H_2_O_2_. EBs at day 4 of differentiation were treated with 1000 nM H_2_O_2_ in the presence of 200 nM MG-132. Twenty-four hours later, cells were collected and subjected for western blot to detect Gata4 protein. **d** Promotion of Gata4 ubiquitination by JNK2 overexpression. P19 cells were transfected with FLAG-Gata4 and His-JNK2 together with HA-tagged ubiquitin. Immunoprecipitation was performed with anti-FLAG and subsequently immunoblotting analysis was conducted with anti-HA antibodies to detect Gata4 ubiquitination. Experiments were repeated three times. **e** Proposed model outlining the role of caspase-3 in activating Gata4 transcription via depleting Nanog and Hdac4

Herein, we further explored the mechanisms underlying H_2_O_2_-induced Gata4 degradation. Firstly, we found that antagonism of JNK cascade but not p38 reversed the downregulation of Gata4 protein at the presence of 1000 nM H_2_O_2_ (Fig. 8a). Next, we measured the half-life of Gata4 protein after combined transfection with JNK2 plasmid in P19 cells. After transfection, cells were treated with the protein synthesis inhibitor cycloheximide (CHX) and subjected to lysis at indicated time points for the detection of Gata4 degradation. As shown in Fig. 8b, JNK2 overexpression accelerated the degradation of Gata4. Consistent with this result, the proteasome inhibitor MG-132 also protected Gata4 protein from H_2_O_2_-induced degradation (Fig. 8c). It implicates that JNK cascade degrades Gata4 protein in a proteasome-dependent manner. Furthermore, we evaluated the effect of JNK2 on Gata4 ubiquitination. As shown in Fig. 8d, forced expression of JNK2 greatly evoked Gata4 ubiquitination compared with vector control. Taken together, our findings indicate that Gata4 is a sensitive factor in response to varying intensity of oxidative stress.

## Discussion

In this study, we revealed the dose-associated effects of ROS on cardiomyogenesis. Mild magnitude of stress-producing stimuli can deplete Nanog and Hdac4 to activate cardiac gene program. However, severe stimuli can degrade Gata4 to interrupt cardiac differentiation.

ROS can act as signaling molecules that extensively participate in the modulation of various cellular events. The effects of ROS are dependent on its intensity and cellular context. In cardiac differentiation, endogenous ROS exerts an indispensable effect. As ROS scavengers such as N-acetyl-cysteine (NAC), catalase, trolox, and pyrrolidine dithiocarbamate, all interrupted the competency of ES cells acquiring cardiac commitment^4–6^. In this study, three stress-producing stimuli, H_2_O_2_, autophagy inducer rapamycin, and ER stress inducer tunicamycin were found to activate cardiomyogenesis via ROS pathway. Relatively, H_2_O_2_ administration at the early stage of differentiation can produce more prominent effects than that at the late stage. The formation stage of EBs is a sensitive period for receiving ROS signals and modifying cell fates. Intriguingly, the expression of Nox4 reaches the peak at the late maturation stage of cardiac differentiation. Thus, the redox and differentiation of stem cells is more important in mediating the effects of ROS.

It was well studied that ROS induced the expression of cardiac-specific genes and transcription factors *Gata4*, *Nkx2.5*, *Mef2C*, as well as differentiation-promoting cytokine *Bmp-10*^6^. Indeed, a previous study has indicated that ROS could trigger the redox activation of c-Jun to upregulate *Gata4*^5^. Another study showed that ROS evoked p38 activation and nuclear translocation of Mef2C^4^. Moreover, ROS is found to induce the activation of PI3K and Jak/STAT and the nuclear translocation of NF-κB, thereby favoring cardiac differentiation^7,14^. Despite that, it is difficult to explain why the temporal treatment of H_2_O_2_ can generate a long-lasting influence on cardiac gene program. In this study, we found that H_2_O_2_ treatment can deplete Nanog and Hdac4, hence liberate the repression on *Gata4* and *Nkx2.5* transcription. Histone deacetylation is a repressive modification indicative of gene silence, and histone acetylation is frequently associated with active transcription. Given that Hdac4 alters the epigenetic status of target genes via deacetylating histones, Hdac4 depletion may persistently affect gene expression. Therefore, our results established a link of H_2_O_2_ treatment with the stable and heritable alterations on gene expression in cardiomyogenesis.

Notably, we found that active caspase-3 was required for the ROS-mediated effects. Two inhibitory factors for cardiac differentiation, Nanog and Hdac4, were eliminated via caspase-dependent pathway. The spatio-temporal activation of caspases takes responsibility to eliminate a limited number of selected substrates, thereby reconstructing cell differentiation status. For instance, caspase-3 cleaves and activates rho-associated kinase-1 (ROCK-1), and consequently triggers cytoskeletal rearrangement, which is indispensable for macrophage polarity and dendrite pruning during neurogenesis^15,16^. Indeed, previous studies definitely indicated that there exists a temporary activation of caspase-3 to deplete Nanog when ES cells entering differentiation status from renewal one^17,18^. In this study, our results showed that the spontaneous activation of caspase-3 occurred at the early stage of cardiac differentiation. Although ROS-activated p38 and JNK kinases enhanced the activation of caspase-3, the driving force of caspase-3 activation seemed to not be p38 or JNK cascades. Because p38 and JNK kinases reach the optimal activities at the late differentiation stage, and at the same time the cleavage of caspase-3 disappears reversely. Thus, we convinced that caspase-3 activation after the onset of cell differentiation is helpful for cell exit from the pluripotent and renewal status, but the trigger mechanism of caspase-3 activation remains obscure. Even so, the activation of caspase-3 at the early stage might lay a foundation for the action of ROS stimulation as the agreement of both in time-window.

A core set of transcription factors cooperates together to maintain the pluripotent state of stem cells. The triumvirate of Oct4, Sox2, and Nanog, predispose the expression of genes governing self-renewal and simultaneously repress genes driving differentiation. In this study, we found that Nanog rather than Oct4 plays a crucial role in establishing the transcriptional silence of *Gata4* and *Nkx2.5*. It has been found that Gata4 can impair the reprogramming transition of fibroblasts from the initiation phase to the iPS status via repressing *Nanog* expression^19^. Herein, our results suggested that Nanog functionally synergizes with Hdac4 to maintain the transcriptional silence of *Gata4* and *Nkx2.5* in stem cells. Previous studies showed that Hdac4 inhibition induce the entry of mesodermal cells into the cardiac muscle lineage, companied by the upregulation of *Gata4* and *Nkx2.5* transcripts^20,21^. It was found that Hdac4 inhibition facilitated myocardial differentiation of progenitor cells in infarcted hearts and ameliorated the restoration of cardiac function^22^. Meanwhile, Hdac4 is also sensitive to caspase-3-mediated cleavage just like Nanog. By comparison, it was found that caspase-3 hardly cleaves Hdac1, 2, 3, 5, and 6^12,13^. Based on the similar feature and function of Nanog and Hdac4, we speculated that Hdac4 is a cofactor of Nanog. Herein, our results showed that Nanog can recruit Hdac4 onto the promoters of *Gata4* and *Nkx2.5* to silence their expression. However, the mechanism of functional collaboration between Nanog and Hdac4 is still poorly understood and need more investigation.

Overall, this study reveals that oxidative stress can deplete Nanog and Hdac4 via a caspase-dependent manner, thereby alleviating the repression on *Gata4* and *Nkx2*.5 promoters and favoring cardiac commitment (Fig. 8e). Meanwhile, our study also indicates that intensive stress can activate JNK cascade to degrade Gata4 protein in a proteasome-dependent manner. Thus, stress-producing stimuli exert biphasic and antagonistic effects on cardiac differentiation dependent on its intensity and duration.

## Materials and methods

### Cell culture and cardiac differentiation

P19 embryonal carcinoma cells were cultured in Dulbecco’s Modified Eagle Medium (DMEM) containing 15% heat-inactivated Fetal Bovine Serum (FBS) (Hyclone, Waltham, MA, USA), 1% non-essential amino-acids (Invitrogen, San Diego, CA USA), 1 mM pyruvate, 2 mM Glutamine, 0.1 mM β-mercaptoethanol in a humidified 5% CO_2_ atmosphere at 37 °C. P19 cells (5 × 10^5^ cells/ml) were placed into 60-mm bacterial grade plastic dishes in the presence of 1% DMSO (Sigma-Aldrich, St. Louis, MO, USA) to initiate cell aggregate and cardiac differentiation. The time point for forming EBs was taken as day 0. At day 4 of cardiac induction, EBs were plated on tissue culture-grade dishes for adherent culture. Spontaneous contractions of EBs were observed from day 8 and the ratio of EBs with beating to the total number of plated EBs was counted.

Total RNA was extracted using Trizol reagent (Invitrogen, San Diego, CA USA). Two micrograms of total RNA from each sample was used for reverse transcription, the PCR was carried out as previously descripted^23^. For real-time reverse transcription polymerase chain reaction (RT-PCR), SYBR Green real-time Master Mix (Toyobo, Japan) and the ABI 7900 Real-Time PCR were used (Applied Biosystems, Foster City, CA, USA). Target gene expression levels were normalized by calculating the Targets/18S expression ratio (2-ΔΔCt). Primers for RT-PCR and real-time RT-PCR are listed in the supplemental information, Table S1.

### Western blot

Western blotting has been descripted as previously reported^23^. Antibodies against Oct4, Sox2, Nanog, Gata4, Nkx2.5, Hdac4, and actin were obtained from Santa Cruz Biotechnology (Dallas, TX, USA); caspase-3, p-p38, p38, and JNK were purchased from Cell Signaling Technology (Danvers, MA, USA); FLAG, HA, and α-actinin antibodies were from Sigma-Aldrich (Sigma-Aldrich, St. Louis, MO, USA). Antibody specific for the sarcomeric MHC (MF-20; Developmental Studies Hybridoma Bank) was generously provided by Dr. Qin Lu (Key Laboratory of Molecular Cardiovascular Sciences, Peking University, China). The figures shown are representative of at least three independent experiments.

### Immunofluorescence

For immunofluorescence identification of differentiated cardiomyocytes, EBs induced until day 12 were digested and replaced on glass coverslips as previously reported^23^. The percentage of α-actinin-positive cells out of the total number of cells counted represented the differentiation efficiency. To display the subcellular localization of Nanog and Hdac4, undifferentiated P19 cells were stained with primary antibody against these two proteins, followed by overnight incubation with goat anti-rabbit FITC and donkey anti-mouse TRITC fluorescence-conjugated secondary antibodies (Santa Cruz, Dallas, TX, USA).

### Plasmids transfection

P19 cells were seeded into 12-well plates 24 h before transfection. Constructs noted in figure legends were transfected using Lipofectamine 2000 (Invitrogen, San Diego, CA USA) according to the manufacturer’s instruction. The plasmid encoding Oct4, Nanog, and FLAG-Hdac4 were kindly provided by Dr. Yinan Liu (Stem Cell Research Center, Peking University Health Science Center). His-JNK2 plasmid was purchased from Sino Biological (Beijing, China). After 24 h of transfection, the cells were digested into single cells and then cultured in suspension to induce cardiac differentiation with 1% DMSO. All experiments were done in triplicates and performed at least three times.

### Chromatin immunoprecipitation (ChIP) assays

ChIP assays were performed on these cells using the EZ ChIP kit (Millipore, Los Angeles, CA, USA) according to the manufacturer’s instructions. Briefly, alter cells were collected and the cross-linked chromatin was sheared to an average DNA fragment length of 100–800 bps, immunoprecipitation was performed using 5 μg of Nanog, Hdac4, or acetyl-histone H3K9 antibodies. Normal IgG was used as a negative control. The precipitated DNA was amplified by RT-PCR or real-time PCR. Primers used to amplify the promoters of target genes as following: *Gata4* F: CTAAATGCCCAATTCCAG, R: CGACACTTCAGTCCCTCA; *Nkx2.5* (S1 region) F: TCAACTCTGGAAGCCCTTAT, R: AGGGTCCTGGGAGTCCTGTT; *Nkx2.5* (S2 region) F: AGCCAGACGAAGAGCAGA-3, R: AGACAGGCAGCGTTATCC.

### Immunoprecipitation

Briefly, P19 or HEK293T cells were cultured in DMEM containing 15% heat-inactivated FBS (Hyclone, Waltham, MA, USA), after transfection cells were lysed in modified RIPA buffer for 30 min at 4 °C with rotation. Two-hundred milligram of cell lysate was pre-cleared with Protein G-Sepharose (Roche, Indianapolis, IN, USA) for 1 h, and then was incubated with anti-FLAG conjugated agarose beads (Sigma-Aldrich, St. Louis, MO, USA) overnight at 4 °C with rotation. Agarose beads were washed with TBS buffer and denatured samples were separated by sodium dodecyl sulfate polyacrylamide gel electrophoresis electrophoresis. Western blot was performed as described above.

### Flow cytometry

The α-actinin and the rabbit IgG isotype control antibodies were purchased from Sigma (Sigma-Aldrich, Pudong, Shanghai, China), the secondary FITC conjugated secondary antibody was purchased from Invitrogen (ThermoFisher Scientific, Carlsbad, CA, USA). Briefly, P19 cells were induced to beating cardiomyocytes and treated as designed, then trypsined to solo suspension cells, after Fc blocking cells were incubated with primary or secondary antibodies in 3% BSA/PBS for 30 min at room temperature in the dark, finally cells were washed three times and resuspended in 500 µl cold PBS and analyzed using a FACS Calibur flow cytometer (BD, Franklin Lakes, NJ, USA).

### Statistical analysis

All data were expressed as the mean ± SD from at least three independent biological replicates. The unpaired Student’s *t*-test or one way ANOVA with significance set at *p* < 0.05 was used to determine statistical significance for each assay. Error bars indicate standard deviation.

## Electronic supplementary material

Supplemental table 1
