# Supplementary material for: Dural effects of oxidative stress on cardiomyogenesis via Gata4 transcription and protein ubiquitination
Source: Cell Death Dis. 2018 Feb 14;9(2):246. doi: 10.1038/s41419-018-0281-y (PMC5833852; doi:10.1038/s41419-018-0281-y)
Supplement: Supplementary file 1 — Supplemental table 1 [file 41419_2018_281_MOESM1_ESM.doc]

Table S1 Primers used for RT-PCR and real-time PCR

| **Gene** | **Primer Sequence(5’ to 3’)** | **AT(°C)** | **PS(bp)** |
| --- | --- | --- | --- |
| *Oct4* | F-AGTTGGCGTGGAGACTTTGC  R-CAGGGCTTTCATGTCCTGG | 58 | 160 |
| *Nanog* | F-CCTGATTCTTCTACCAGTCCCA  R-GGCCTGAGAGAACACAGTCC | 58 | 123 |
| *Gata4* | F-CACCCCAATCTCGATATGTTTGA  R-GGTTGATGCCGTTCATCTTGT | 58 | 151 |
| *Nkx2.5* | F-CAAGTGCTCTCCTGCTTTCC  R- GGCTTTGTCCAGCTCCACT | 56 | 136 |
| *α-Mhc* | F- GCCCAGTACCTCCGAAAGTC  R- GCCTTAACATACTCCTCCTTGTC | 58 | 110 |
| *β-Mhc* | F- ACAACCCCTACGATTATGCGT  R- ACGTCAAAGGCACTATCCGTG | 58 | 100 |
| *18S rRNA* | F- GTAACCCGTTGAACCCCATT  R-CCATCCAATCGGTAGTAGCG | 58 | 151 |
